# Importance of numerical density of tubulointerstitium infiltrates in the prognosis of antineutrophil cytoplasmic antibodies-associated glomerulonephritis

**DOI:** 10.3325/cmj.2023.64.37

**Published:** 2023-02

**Authors:** Dušan Božić, Bojana Ljubičić, Violeta Knežević, Dejan Ćelić, Vladimir Veselinov

**Affiliations:** 1 Medical Faculty, University of Novi Sad, Novi Sad, Serbia; 2 Clinic of Nephrology and Clinical Immunology, University Clinical Center of Vojvodina, Novi Sad, Serbia; 3 Department of Emergency Internal Medicine, University Clinical Centre of Vojvodina, Novi Sad, Serbia

## Abstract

**Aim:**

To examine the association of the numerical density of the tubulointerstitium infiltrate with pathohistological changes in the glomeruli and the estimated glomerular filtration rate (eGFR) at kidney biopsy and after 18 months.

**Methods:**

This retrospective study enrolled 44 patients (43.2% male) with antineutrophil cytoplasmic antibodies-associated glomerulonephritis treated at the University Clinical Center of Vojvodina between 2017 and 2020. The numerical density of infiltrates in the tubulointerstitium was determined with the Weibel (M-2) system. Data on biochemical, clinical, and pathohistological parameters were obtained.

**Results:**

The mean age was 57.7 ± 10.23 years. Global sclerosis in more than 50% of glomeruli and crescents in more than 50% of glomeruli were significantly associated with a mean lower eGFR (17.6 ± 11.78; 32.0 ± 26.13, respectively) at kidney biopsy (*P* = 0.002; *P* < 0.001, respectively), but not after 18 months. The average numerical density of infiltrates was significantly higher in patients with more than 50% of globally sclerotic glomeruli (*P* < 0.001) and with crescents in more than 50% of glomeruli (*P* < 0.001). The average numerical density of infiltrates significantly correlated with eGFR at biopsy (r = -0.614), but not after 18 months. Our results were confirmed by using multiple linear regression.

**Conclusion:**

Numerical density of infiltrates, and global glomerular sclerosis and crescents in more than 50% of glomeruli significantly affect eGFR at biopsy, but not after 18 months.

Primary antineutrophil cytoplasmic antibodies (ANCA)-associated vasculitides (AAV) are a group of autoimmune chronic multisystem diseases including microscopic polyangitis, granulomatosis with polyangitis, better known as Wegener's granulomatosis, and eosinophilic granulomatosis with polyangitis. These disorders are treated with corticosteroids and immunosuppressive therapy and have an indefinite prognosis closely linked to a timely diagnosis (1). In recent years, their prevalence has increased as a result of better diagnostic possibilities.

Among the organs most frequently affected by these diseases are the kidneys. Kidney involvement deteriorates the clinical course and indicates a severe form of the disease. Kidney changes are often manifested as rapidly progressive glomerulonephritis with a serious prognosis, and they can permanently reduce kidney function, requiring an intensive therapy and dialysis (2). Untreated patients with AAV and kidney involvement have a mortality rate of up to 80%. However, patients who undergo an aggressive immunosuppressive therapy have a five-year survival rate of about 75% (3,4). In addition to kidney manifestations, the prognosis of AAV is impaired by the occurrence of pulmonary hemorrhage, early need for dialysis, and old age (5).

Glomerular changes appear in the form of extensive crescent glomerulonephritis, while immunofluorescence microscopy shows a pauci-immune pattern. Pathohistological changes in the glomeruli are classified according to their frequency and extent, crescent count, and the presence of glomerular sclerosis (6,7). Vasculitis is the cause of rapidly progressive glomerulonephritis in about 80% of the cases (2).

In addition to the basic changes in the glomeruli, which are characteristic of secondary glomerulonephritis, the inflammation process involves infiltrations of the tubulointerstitium, primarily by T-lymphocytes and monocytes (8). Lymphocytes are predominantly located periglomerularly, especially around the glomeruli with crescents and sclerotic glomeruli. Additionally, monocytes, neutrophils, and even plasma cells may be diffusely located along the tubulointerstitium (9,10). Tubular atrophy and interstitial fibrosis are also observed, especially in the presence of a large number of CD-68 positive monocytes or macrophage cells (11). When changes to the glomeruli are severe, basement membranes of the glomeruli rupture and their contents are discharged into the Bowman space. This is a major trigger for pronounced tubulointerstitial infiltration observed in patients with a poor prognosis and rapidly deteriorating kidney function (12,13). Some authors believe that tubulointerstitial infiltration occurs as an independent phenomenon, primarily under the influence of interleukin-1β (14). Therefore, some argue that diffuse infiltration by lymphocytes and monocytes is an independent parameter for a poorer prognosis for the kidneys (15). Infiltration precedes tubular atrophy and interstitial fibrosis, which have already been proven to confer a significantly greater risk for a terminal stage of kidney disease (16-18). The aim of the study was to examine the association of the tubulointerstitial infiltrate density with pathohistological changes in the glomeruli and the estimated glomerular filtration rate (eGFR) at kidney biopsy and after 18 months.

## Patients and methods

This retrospective study enrolled 44 patients with AAV and secondary AAGN. All patients were diagnosed with vasculitis with kidney involvement. Kidney function was determined, and a percutaneous kidney biopsy was performed, which was the beginning of follow-up. The diagnosis was based on the Chapel Hill Consensus Conference Criteria for ANCA-associated vasculitis (19). We collected demographic, biochemical, and pathohistological parameters and quantitatively determined cell infiltrate density in the tubulointerstitium. Kidney function was verified by determining serum creatinine and eGFR calculated according to the Cockcroft-Gault formula.

All patients were treated with corticosteroids and cyclophosphamide. Twenty patients with a rapidly progressive disease course, extrarenal manifestations, and creatinine level over 500 umol/L were also treated with plasmapheresis and, when necessary, renal replacement therapy. Immunosuppressive therapy was applied on the basis of the recommendations of the European Kidney Association – European Dialysis and Transplant Association. Cyclophosphamide was administered according to the National Institute of Health protocol (20), with the dose ranging from 500 to 1000 mg/m^2^ depending on the kidney function. Methyl-prednisolone therapy began with a three-day pulse therapy of 7 mg/kg/TT per day, then 1 mg/kg/TT for three weeks, then orally at the same daily dose, with a successive reduction of daily dose (by about 25% every 4 weeks) in the course of minimum three months. The follow-up period lasted 18 months.

Kidney samples were obtained by percutaneous kidney biopsy by using 1.6-mm diameter Tru-Cut needles. The samples were fixed in alcohol (70%), stained with hematoxylin and eosin, and embedded in paraffin. Patients with fewer than 10 glomeruli were not included in the study. To determine the numerical density of the infiltrate, the material was cut on a 5-micron thick microtome. The cells were counted in every other incision to avoid re-counting the same cells.

Numerical density is a relative stereological quantity that is defined as the number of particles in a unit of volume. We used the so-called thick incision, because the cell size in our study was significantly smaller than the thickness of the incision. To determine the numerical density of thick incisions we used Abercrombie's method. We counted the cells with the center in the section itself, as well as those whose center was in the layer above or below the section, but was partially affected. We used the formula NVF = NA / (t+D), where NVF is the numerical density, NA is the number of cells in the cross section, t is the thickness of the cut, and D is the average cell diameter.

**Figure 1:**
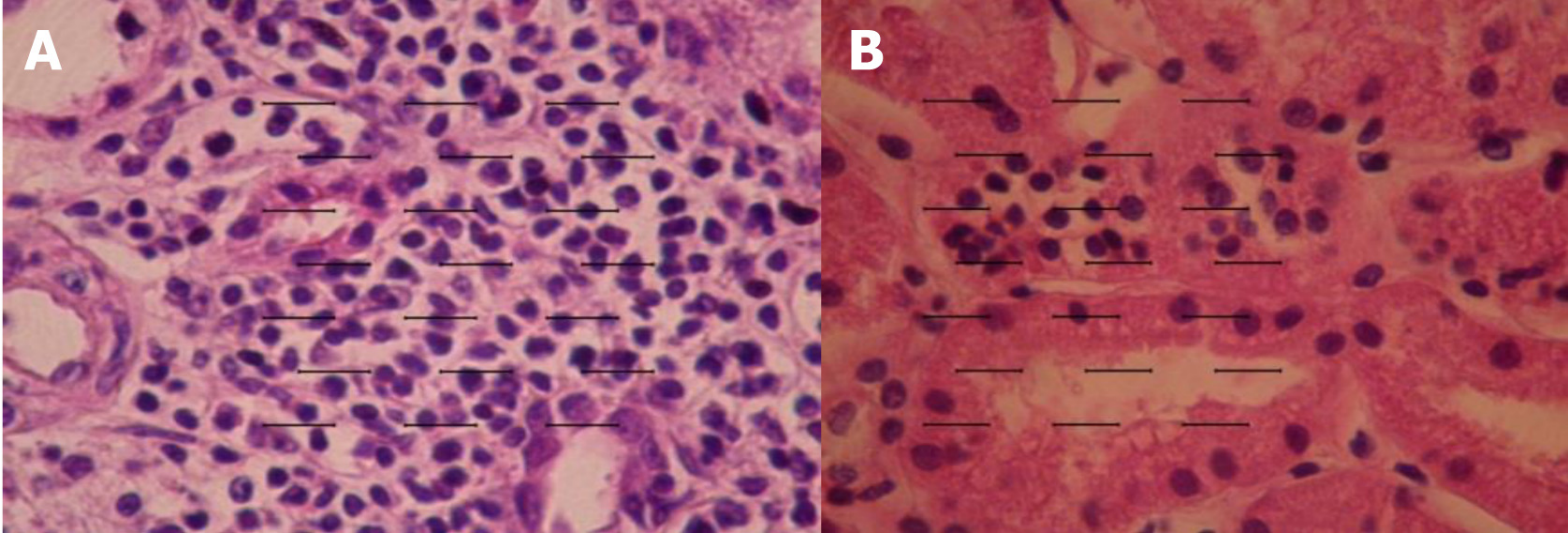
Kidney interstitium with a high (**A**) and low (**B**) numerical density of infiltrates. Observed with a microscope with a built-in Weibel 42 system.

Cells were counted by using the multifunctional M42 system according to Weibel et al (21), which was introduced in order to avoid a large number of cross-sections or contours of the tested structure with test lines. The number of test points that can be targeted is 42. The advantage of the system is clear visibility during counting, even when the grid does not cover the entire viewing field. This means that only the targeted points of the system are counted, and their number is added to the calculation formula (Figure 1).

Numerical density was assessed at a magnification of 400 × , with an incorporated “grid” or test system in the eyepiece (Carl Zeiss GF-Pw 10 × , Carl Zeiss, Jena, Germany). A video-camera was incorporated into the microscopic system. In both cases, the calibration distance between two adjacent lines was 1.04 or 2.08 microns (21,22). The numerical density of the infiltrate was measured periglomerularly around the glomeruli (3 counts) and interglomerularly in sites away from the glomeruli (3 counts), ie, in a total of 6 visual fields. All cells in the infiltrate were counted (lymphocytes, monocytes/acrophages and plasma cells, fibrocytes, and polymorphonuclear granulocytes).

Numerical infiltrate density was compared with eGFR at the beginning and end of the study, the presence of glomerular crescents, and the presence of glomerular sclerosis (<50%/≥50%).

### Statistical analysis

Data are presented as means and standard deviations. *t* test with the level of significance of *P* ≤ 0.05 was used to assess the significance of differences between the groups. Pearson correlation coefficient was used to assess the correlation of two continuous variables (0.6-0.8 – high correlation; 0.8-1.00 – very high correlation). Multiple linear regression was also applied. Predictors were variables that showed significant differences or correlation with eGFR *t* = 0. Statistical analysis was performed with STATISTICA Science Workbench 14.0 software (Tibco, Palo Alto, CA, USA).

## RESULTS

The study enrolled 44 patients (19 or 43.2% men) with pathohistologically verified ANCA-associated glomerulonephritis. The mean age at the time of diagnosis was 57.7 ± 10.23 years. Positive anti-MPO antibodies were present in 18 (41%) and positive anti-PR3 antibodies in 26 patients (59%) (Table 1).

**Table 1:** Demographic, clinical, and pathohistological characteristics*

Patients	n = 44
Men, n (%)	19 (32.2)
Age (years), mean	57.7 ± 10,23
**Anti-neutrophil cytoplasmatic antibody type, n (%)**	
proteinase 3	26 (59)
myeloperoxidase	18 (41)
**Pathohistological subgrouping, n (%)**	
sclerotic class	24 (54.5)
crescentic class	20 (45)
**Numerical density of the infiltrate** ( **× mm^−3^), mean ± standard deviation**	65 109.91 ± 32.52
around the glomeruli	71 678.36 ± 35943.12
in the wider space among glomeruli	58 541.45 ± 29448.08

At the time of diagnosis, the mean eGFR in patients was 53.27 ± 43.25 mL/min/1.73m^2^, and after 18 months of treatment it was 59.43 ± 37.84 mL/min/1.73m^2^. In patients with anti-MPO antibodies, the mean eGFR was significantly lower (32.89 ± 28.27 mL/min/1.73m^2^) than in patients with AAGN (69.20 ± 46.57 mL/min/1.73m^2^; *P* = 0.005). After 18 months, the mean eGFR in patients with anti-MPO antibodies was 46 ± 23.97 mL/min/1.73m^2^ and in patients with anti-PR-3 antibodies it was 68.73 ± 43.05 mL/min/1.73m^2^. There was no significant difference in eGFR between patients with different type of ANCA antibodies at the beginning (*P* = 0.0054) and end of the study (*P* = 0.1288).

At the time of diagnosis, more than 50% of glomerular crescents were found in 24 (54.5%) patients. Patients with more than 50% glomerular crescents had a significantly lower mean eGFR (32.0 ± 26.13 mL/min) than patients with less than 50% glomerular crescents (78.8 ± 46.37 mL/min (*t* = -4421, *P* = 0.0001). More than 50% of globally sclerotic glomeruli were found in 10 (22.7%) patients. These patients had a significantly lower mean eGFR (17.6 ± 11.78 mL/min/1.73m^2^) than the group with less than 50% of sclerotic glomeruli (63.8 ± 43.6 mL/min; *t* = 3288, *P* = 0.002). The change in eGFR (difference between eGFR at the beginning and after 18 months) was not associated with the percentage of crescents in glomeruli (*t* = 0.7405 *P* = 0.463). Also, there was no significant association between the change in eGFR and the percentage of sclerotic glomeruli (*t* = 0.6568, *P* = 0.515) (Table 2).

**Table 2:** Estimated glomerular filtration rate (eGFR) in relation to histopathological subgroup at the time of biopsy and after 18 months*

	eGFR at the time of biopsy	p	eGFR after 18 months	p
Glomerular sclerosis				
≥50%	17.6 ± 11.78	0.002	+1.10 ± 6.72	0.515
<50%	63.8 ± 43.60		+7.35 ± 29.65	
Crescentic glomeruli				
≥50%	32.0 ± 26.13	0.0001	+8.62 ± 23.85	0.463
<50%	78.8 ± 46.37		+2.70 ± 29.24	

The numerical density of infiltrate in the tubulointerstitium was determined in all patients. The average density of infiltrates was 65 109.91 ± 32.52 × mm^−3^. The mean density of infiltrates was significantly higher around the glomeruli (71,678.36 ± 35 943.12 × mm^−3^) than in the wider space among the glomeruli (58,541.45 ± 29 448.08 × mm^−3^; *t* = 9.29, *P* < 0.0001). The average numerical density of infiltrates was significantly higher in patients with crescents in more than 50% of glomeruli (80,422.69 ± 28 786.4 × mm^−3^) than in patients with crescents in less than 50% of glomeruli (46,734.58 ± 27 178.8 × mm^−3^; *t* = 3.96, *P* < 0.001). The average numerical density of infiltrates was significantly higher (10,1677.4 ± 27 727.6 × mm^−3^) in patients with more than 50% of globally sclerotic glomeruli than in patients with less than 50% of sclerotic glomeruli (54,354.76 ± 25 376.4 × mm^−3^; *t* = -5.079, *P* < 0.0001). Regardless of the infiltrate localization, there was no significant difference in infiltrate densities between patients with anti-PR3 and those with anti-MPO antibodies (Table 3).

**Table 3:** Numerical density of infiltrates in the interstitium in relation to histopathological subgroup and the type of antibody

	Numerical density of infiltrates (Number of cells x mm-3)	p
Glomerular sclerosis		
≥50%	101677.4 ± 27727.6	<0.0001
<50%	54354.8 ± 24376.4	
Crescentic glomeruli		
≥50%	80 422.7 ± 28786.4	0.0003
<50%	46 734.6 ± 27178.8	
**ANCA type**		
anti- proteinase 3 antibodies	359897.0 ± 34833.3	0.362
anti- myeloperoxidase antibodies	72639.7 ± 28089.8	

**Figure 2:**
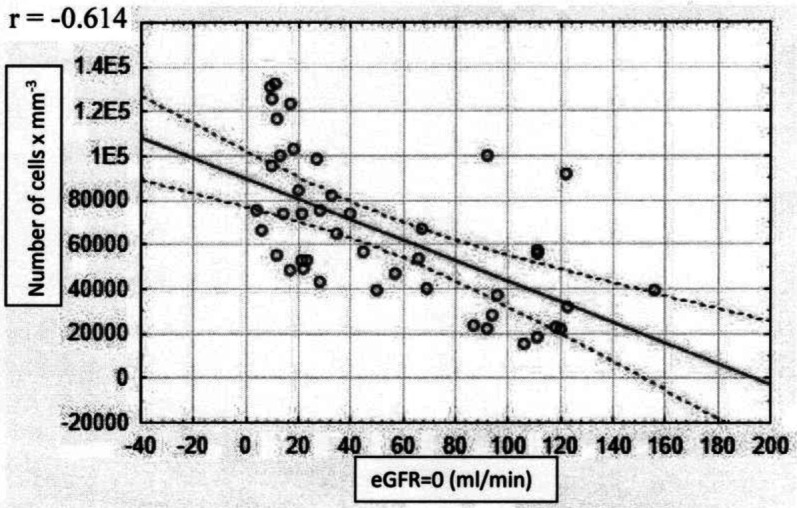
Correlation of numerical density of interstitial infiltrate and estimated glomerular filtration rate (eGFR) at the time of biopsy.

**Figure 3:**
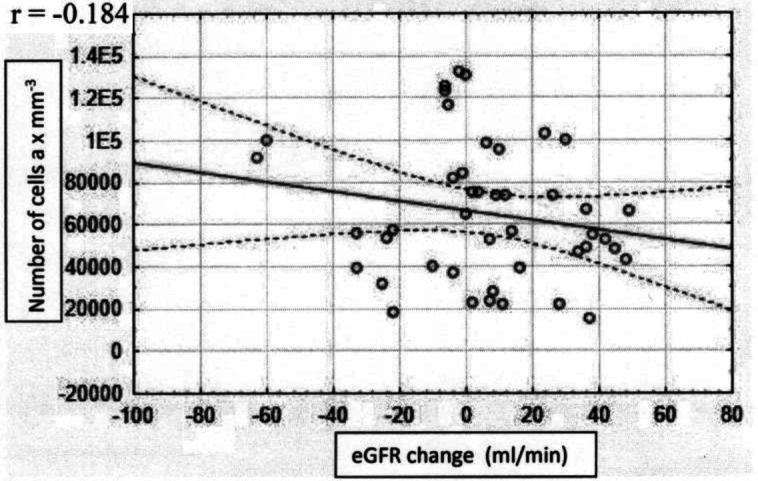
Correlation of numerical density of interstitial infiltrate and change of estimated glomerular filtration rate (eGFR) after 18 months.

The numerical density of interstitial infiltration significantly correlated with eGFR at biopsy (r = -0.614) (Figure 2), but it did not correlate with changes in eGFR (r = -0.1842) (Figure 3).

The mean numerical density of interstitial infiltration (β = -0.100; *P* = 0.028), presence of ≥50% crescents in glomeruli (β = -26.905; *P* = 0.03), and presence of ≥50% globally sclerotic glomeruli (β = -12.213; *P* = 0.042) significantly negatively affected eGFR at the time of biopsy (Table 4). This linear regression model explained 44% variety of eGFR at the time of biopsy.

**Table 4:** The impact of numerical density of infiltrates in the interstitium, global sclerosis, and the presence of crescents on estimated glomerular filtration rate (eGFR) at the time of biopsy

Dependent variable GFR *t* = 0	β	Std. Gr.	t	p	-95% confidence interval	+95% confidence interval
Constant	101.785	12.081	8.425	0.000	77.369	126.202
Interstitium infiltrate	-0.100	0.071	-2.281	0.028	-0.384	-0.027
Crescent ≥50%	-26.905	11.942	-2.253	0.030	-51.041	-2.769
Sclerosis of glomeruli ≥50%	-12.213	15.030	-1.813	0.042	-62.590	-1.164

A linear regression model (F (3,40) = 1.455; *P* = 0.241; R2 = 0.439) showed that the average density of the infiltrate, the presence of more than of 50% crescents in glomeruli, and the presence of more than of 50% of globally sclerotic glomeruli did not significantly affect the changes in eGFR (Table 5).

**Table 5:** The impact of numerical density of infiltrates in the interstitium, global sclerosis and the presence of crescents on change of estimated glomerular filtration rate (eGFR) after 18 months

Dependent variable GFR change	β	Std. Gr.	t	p	-95% CI	+95% CI
Constant	15.180	9.307	1.631	0.111	-3.629	33.990
Interstitium infiltrate	0.000	0.000	-1.644	0.108	-0.001	0.000
Crescents ≥50%	15.160	9.200	1.648	0.107	-3.433	33.753
Sclerosis of glomeruli ≥50%	-1.215	11.579	-0.105	0.917	-24.616	22.186

## DISCUSSION

In this study, the numerical density of infiltrates, and global glomerular sclerosis and crescents in more than 50% of glomeruli significantly affected eGFR at biopsy, but not after 18 months. The extensiveness of glomerular changes is important for disease prognosis. Also, many authors point to the impact of the changes in the tubulointerstitium (mononuclear cell infiltration, tubular atrophy, and fibrosis) on the deterioration of kidney function (13,23).

AAV mostly occurs in the elderly population, but the prevalence in younger age groups has been increasing. The average age of our patients was similar to that in other studies on AAGN (12). In most patients, eGFR was already reduced at the time of diagnosis (less than 60mL/min/1.73m^2^). This indicates that the diagnosis of AAV was made late, as is often the case as these patients suffer from polymorphic disorders and oligosymptomatic manifestations of the disease (24). This also explains the significantly lower eGFR in patients with anti-MPO antibodies, who have a higher frequency of respiratory symptoms and changes, compared with patients with anti-PR3 antibodies. In any case, eGFR improved after 18 months of follow-up, although not significantly so. We can assume the kidney function did not deteriorate further because patients received therapy. Immunosuppressive therapy has been shown to offer benefits for survival in these patients and to slow down the decline of kidney function (3,5).

In 54.5% of patients, crescents were found in more than 50% of the glomeruli. These patients had a significantly lower eGFR compared with the group with less than 50% crescents. However, this difference was not found at the end of the follow-up. Similar results were obtained in 22.7% of patients with a significant presence of globally sclerotic glomeruli. At the beginning of the study, these patients had a significantly lower eGFR compared with the group with less than 50% of globally sclerotic glomeruli. We noticed no significant change in kidney function between the beginning of the follow-up and after 18 months. Even in patients with more than 50% of sclerotic glomeruli, there was no significant deterioration in kidney function with the prescribed therapy. The higher incidence of crescents and global sclerosis has previously been recognized as histological changes associated with lower kidney function (24,25). Infiltrate densities were equal regardless of the antibody type (anti-PR3 or anti-MPO) and there was no significant difference between patients according to this parameter.

The average density of infiltrates in the tubulointerstitium was 65 109.91 × mm^−3^. The infiltrate density was significantly higher around the glomeruli than in other parts of the tubulointerstitium. Other studies also noted this difference (9). The numerical density of infiltrates was significantly higher in patients with the presence of crescents in more than 50% of glomeruli and in patients with more than 50% of globally sclerotic glomeruli. We observed a high correlation of infiltrate densities and eGFR at the time of biopsy, which means that eGFR was significantly lower in patients with higher interstitial infiltrate densities. The high correlation was present regardless of the infiltrate location. However, no significant correlation of the dynamics of kidney function during the study (decrease/increase in eGFR) and numerical density of the infiltrate was found. The multiple linear regression model confirmed the results of the influence of the numerical density of the infiltrate, global glomerular sclerosis, and the presence of crescents in more than 50% of the glomeruli on eGFR at the time of biopsy and after 18 months. Other studies found that the presence of crescents in more than 50% of glomeruli, global glomerular sclerosis, and tubulointerstitial infiltration were correlated with initial creatinine and lower GFR, but not with the progression of kidney failure in patients who were treated in the meantime (9,16,26,27).

Our previous study, which enrolled 216 patients with different types of primary glomerulonephritis over a follow-up period of 77.5 months, established a significant association between the numerical density of the infiltrate of all cells and parameters of renal function, independent of the type of glomerulonephritis. Tubulointerstitial infiltration was established as a prognostic predictor for these diseases (28).

A high density of infiltrates in the tubulointerstitium accompanies the most severe histological changes in the glomeruli. This parameter indicates a severe form of AAGN. Patients with less severe tubulointerstitial infiltration more commonly experienced a relapse. This means that these patients must also be followed up periodically with a possibility of intensifying immunosuppressive therapy during a relapse (29).

The limitations of this study were the small sample size and the lack of adjustment for other important prognostic factors for AAV such as proteinuria, antiproteinuric/kidney protective medications, etc. Also, this is a single-center study, so future multi-center studies with greater sample size should be conducted to confirm our findings.

In conclusion, the numerical density of the infiltrate, global glomerular sclerosis, and the presence of crescents in more than 50% of glomeruli significantly affected eGFR at the time of biopsy, but not after 18 months.
